# Human–Machine Collaboration Depth and Teacher Burnout: A Dual-Path Moderated Mediation Model of Empowerment and Depletion

**DOI:** 10.3390/bs16081324

**Published:** 2026-08-03

**Authors:** Xiaoyu Guo, Man Li, Xin Zhao

**Affiliations:** 1 School of Computer Science and Engineering, Northeastern University, Shenyang 110819, China; 2 School of Management, Northeastern University at Qinhuangdao, Qinhuangdao 066004, China; liman@neuq.edu.cn (M.L.); zhaoxin@neuq.edu.cn (X.Z.)

**Keywords:** human–machine collaboration depth, teacher burnout, digital burden, teacher agency, perceived algorithmic control

## Abstract

This study investigates how human–machine collaboration depth is associated with teacher burnout during educational digital transformation. Grounded in Conservation of Resources theory, cross-sectional survey data from 489 Chinese university teachers were analyzed using structural equation modeling and conditional process analysis. Results indicated that human–machine collaboration depth was negatively associated with teacher burnout, mediated in parallel by digital burden and teacher agency. Within the present sample, the indirect inverse association via teacher agency was numerically larger in magnitude than the positive association via digital burden. Furthermore, perceived algorithmic control acted as a boundary condition. High levels of perceived algorithmic control strengthened the link to digital burden and weakened the protective role of teacher agency. Although the cross-sectional design precludes definitive causal inferences, this study advances a competitive integration perspective of technology use. The findings extend algorithmic management theory to higher education, indicating that mitigating occupational strain requires governance frameworks that prioritize professional autonomy over rigid algorithmic surveillance.

## 1. Introduction

The integration of generative artificial intelligence (GenAI) is reshaping higher education pedagogy, as these advanced systems prompt university educators to fundamentally rethink their instructional approaches (Zaimoğlu & Dağtaş, 2025). While these tools offer cognitive scaffolding for instructional innovation, the rapid pace of digital transformation introduces distinct psychological challenges for educators, highlighting a divergent trajectory of psychological and pedagogical outcomes (Wen et al., 2025). Empirical evidence indicates that technology-induced educational anxiety is linked to the depletion of faculty mental resources (Zhang & Cao, 2025). Accordingly, contemporary teacher burnout no longer stems solely from conventional workloads; it manifests as a specific form of psychological exhaustion tied to technology-driven institutional changes.

The existing literature on educational artificial intelligence has predominantly focused on educators’ initial adoption intentions (Wu et al., 2025) or relied on simplistic metrics such as usage frequency to gauge technology integration (Burton-Jones & Straub, 2006). This approach incorrectly equates mere daily access with effective pedagogical application, leading many institutions to enforce arbitrary adoption metrics that may inadvertently contribute to elevated faculty workload. Such superficial measurements fail to capture the cognitive nuances of human–AI interaction. To address this limitation, Burton-Jones and Straub (2006) conceptualized human–machine collaboration depth (HMCD), defined as the extent to which users cognitively integrate system functionalities into their core work tasks. Evaluating this depth is critical: fragmented tool use is associated with disrupted instructional workflows, whereas internalized collaboration may help optimize cognitive load.

The empirical paradox—where technology integration empowers some educators while exhausting others—calls for a dual-path analytical framework. This study draws on Conservation of Resources (COR) theory and Self-Determination Theory (SDT) to explain this divergence. According to COR theory, navigating complex systems requires continuous algorithmic adaptation, which is linked to digital burden (DB), a construct defined by technology overload and constant connectivity (Tarafdar et al., 2007). This constant adaptation is theorized to drain psychological resources, forming a depletion pathway that is positively associated with teacher burnout. Conversely, SDT posits that deep technology integration can satisfy innate psychological needs by positioning educators as proactive innovators. This process may contribute to greater teacher agency (TA), potentially equipping faculty with a robust defensive resource against workplace strain (Bakker & Demerouti, 2017; Kahn et al., 2025). Yet, how these concurrent empowerment and depletion mechanisms operate in tandem remains empirically under-explored.

This competitive process unfolds within specific organizational climates. In modern higher education, data-driven performance metrics subject faculty to pervasive tracking, which contributes to perceptions of algorithmic control (PAC). When algorithmic surveillance dominates institutional practices, human–AI collaboration risks shifting from an autonomous choice to a compliance tactic (Zhu et al., 2024). Under high levels of such monitoring, deep engagement with technology can become a forced exercise in metric alignment. Such environments are positively associated with digital burden and negatively linked to teacher agency (Röhl, 2025). Despite these implications, few empirical studies have examined how perceived algorithmic control shapes the trajectory of technology-associated workplace strain as a key boundary condition.

To address these gaps, we test a moderated mediation model examining how human–machine collaboration depth is associated with teacher burnout through two competing pathways—digital burden and teacher agency—with perceived algorithmic control acting as a contextual moderator. Drawing on data from 489 university teachers, the study makes two core contributions. First, while constructs such as digital burden, teacher agency, and algorithmic control are well established in neighboring research domains, this work adds unique value by integrating these frameworks to account for divergent psychological outcomes of generative artificial intelligence use among higher education faculty. Second, the findings offer empirical evidence aligned with critiques of technological determinism. Specifically, the results suggest that institutional evaluation structures, alongside the digital tools themselves, conditionally moderate the association between human–machine collaboration depth and educator well-being. This perspective yields actionable insights for administrators seeking to design balanced governance systems.

## 2. Literature Review and Hypotheses

### 2.1. Theoretical Framework

This study integrates COR theory and SDT to elucidate the mechanisms linking technology use to educator well-being. Because a single theoretical lens is insufficient to capture both energetic costs and motivational benefits, two complementary frameworks are employed simultaneously. We draw on COR theory to account for the resource depletion pathway. Rooted in the core premise that individuals actively seek to obtain, retain, and protect valued personal resources (Hobfoll, 1989), this framework outlines how continuous algorithmic adaptation places sustained demands on energetic resources. In turn, SDT frames the motivational gain pathway, explaining how deep human–machine collaboration can satisfy basic psychological needs and support greater teacher agency. COR theory underpins the hypotheses for the depletion pathway via digital burden, whereas SDT grounds the hypotheses for the gain pathway via teacher agency. Furthermore, perceived algorithmic control functions as a boundary condition across both frameworks: it represents an environmental stressor in the COR framework and a need-thwarting organizational context in SDT.

### 2.2. Human–Machine Collaboration Depth and Teacher Burnout

The existing literature on the educational applications of artificial intelligence has predominantly focused on initial adoption intentions or simplistic usage frequencies (Crompton & Burke, 2023). However, prior studies indicate that rudimentary, high-frequency technology use does not automatically yield positive psychological feedback (Zhang & Cao, 2025). To capture the actual psychological impacts of information systems, researchers must examine the qualitative nature of utilization—specifically how users integrate system functionalities into core tasks (Burton-Jones & Straub, 2006). In smart higher education settings, human–machine collaboration depth extends beyond superficial software adoption, representing the extent to which educators cognitively internalize intelligent tools, moving from basic content generation to iterative, collaborative pedagogical co-creation (Kim, 2024).

From a COR perspective, teacher burnout develops from the continuous, unmitigated depletion of valued psychological and energetic resources (Byrne, 1991). When university teachers engage with technology at a superficial level, frequent task-switching and fragmented tool use disrupt cognitive continuity, which is theoretically associated with heightened resource drain. Conversely, an elevated level of human–machine collaboration depth is posited to help educators restructure daily workflows and mitigate cognitive overload. Deep pedagogical co-creation with advanced algorithms yields efficiency gains, enabling faculty to navigate complex instructional and research demands effectively (Dehghan, 2026). Within the COR framework, this workflow optimization serves as a resource acquisition mechanism; the resulting performance gains are linked to lower emotional exhaustion, bolstered personal accomplishment, and a protective buffer against workplace strain. In light of these theoretical deductions, this study hypothesizes the following:

**H1.**  *Human–machine collaboration depth is negatively related to teacher burnout*.

### 2.3. The Mediating Role of Digital Burden

Despite the workflow efficiencies promised by advanced tools, the deep cognitive integration of artificial intelligence within professional tasks requires significant resource expenditure. In information systems research, digital burden denotes the cognitive and psychological overstimulation experienced by employees during intensive technology assimilation, typically manifested as information overload and constant connectivity (Tarafdar et al., 2007). Navigating a high level of human–machine collaboration depth requires university educators to continuously adapt to changing algorithmic architectures and master prompt engineering. This intensive adaptation loop is theoretically associated with technostress and role ambiguity (Högemann et al., 2025), increasing work-related stress during pedagogical practices (Acosta-Enriquez et al., 2025).

Grounded in the COR framework, prolonged resource expenditure without sufficient recovery opportunities aligns with a theorized resource depletion pathway (Hobfoll, 2001). Chronic exposure to high digital burden is theoretically linked to the depletion of emotional reserves and technology-associated educational anxiety (Zhang & Cao, 2025). Furthermore, constant connectivity is associated with blurred boundaries between professional obligations and personal life, which may limit educators’ access to vital psychological restoration (Tarafdar et al., 2007). This sustained draw on energetic resources is consistently associated with symptoms of emotional exhaustion and depersonalization. While human–machine collaboration depth is negatively associated with teacher burnout, digital burden may offset the potential well-being benefits of deeper engagement by functioning as a parallel depletion mechanism. Therefore, we propose the following mediation hypothesis:

**H2.**  *Digital burden mediates the relationship between human–machine collaboration depth and teacher burnout*.

### 2.4. The Mediating Role of Teacher Agency

Conversely, deep engagement with intelligent technology points to a motivational enrichment pathway rooted primarily in SDT (Kim, 2024). Within digital educational ecosystems, teacher agency refers to educators’ capacity to exercise professional judgment and reconstruct instructional identities (Kahn et al., 2025). When university instructors participate in iterative pedagogical co-design with generative AI tools, such interactions may satisfy their core psychological needs for autonomy and competence. Meeting these fundamental needs allows educators to integrate digital affordances into individual teaching practices and reclaim a meaningful sense of professional agency (Aagaard et al., 2022).

From this SDT perspective, teacher agency functions as a motivational asset that helps buffer educators against workplace alienation (Van den Broeck et al., 2016). When professional agency is restricted, educators often experience powerlessness, which relates to depersonalization (Röhl, 2025). In contrast, educators possessing elevated agency maintain cognitive flexibility, suggesting a theoretically plausible protective mechanism against emotional exhaustion. Greater human–machine collaboration depth operates as a catalyst expanding professional agency, suggesting a motivational enrichment pathway that is negatively associated with teacher burnout. Accordingly, this research predicts the following:

**H3.**  *Teacher agency mediates the relationship between human–machine collaboration depth and teacher burnout*.

### 2.5. The Moderating Role of Perceived Algorithmic Control

Although the pathways of resource depletion and enrichment operate concurrently, their relative strength depends heavily on the institutional climate. While the construct of perceived algorithmic control originally described real-time behavior tracking and behavioral constraints in platform economies (Zhu et al., 2024), the implementation of data-driven metrics has introduced an algorithmic climate into contemporary higher education. Algorithmic management in academic settings distinctively focuses on quantifying performance outputs and digitalized evaluation rankings rather than tracking real-time labor processes (Kellogg et al., 2020).

When university teachers operate under intense perceived algorithmic control, human–AI interaction risks becoming alienated from its original purpose. Under high perceived algorithmic control, deep technology integration is no longer driven by autonomous pedagogical curiosity but is coerced by rigid automated metrics and compliance with digital evaluation systems (Pei et al., 2021). This involuntary technology use intensifies role stress and is associated with elevated digital burden, forcing teachers to constantly defend their autonomy against automated assessment systems (Röhl, 2025). Severe administrative monitoring is associated with shifting deep collaboration into a taxing obligation, a dynamic that theoretically corresponds to heightened resource depletion.

When perceived algorithmic control is low, educators retain professional latitude, allowing AI integration to remain a self-directed strategy that may buffer cognitive friction.

Furthermore, drawing on SDT, controlling institutional climates thwart basic psychological needs for autonomy and competence, undermining intrinsic motivation. Under high levels of perceived algorithmic control, the intensive quantification of educational outputs diminishes feelings of self-determination. This administrative control may limit the translation of human–machine collaboration depth into a supportive sense of teacher agency (Kellogg et al., 2020). In contrast, low-control environments respect professional discretion, facilitating conditions where technology use can support greater instructional competence and expanded teacher agency.

Taken together, perceived algorithmic control functions as an environmental moderator. It is hypothesized to strengthen the indirect depletion pathway rooted in COR and weaken the motivational enrichment pathway grounded in SDT. Based on these comprehensive theoretical integrations, the following boundary-condition hypotheses are proposed:

**H4.**  *Perceived algorithmic control moderates the mediating effect of digital burden between human–machine collaboration depth and teacher burnout, such that the indirect resource-depletion effect is stronger under higher levels of perceived algorithmic control*.

**H5.**  *Perceived algorithmic control moderates the mediating effect of teacher agency between human–machine collaboration depth and teacher burnout, such that the indirect resource-enrichment effect is weaker under higher levels of perceived algorithmic control*.

**Figure 1:**
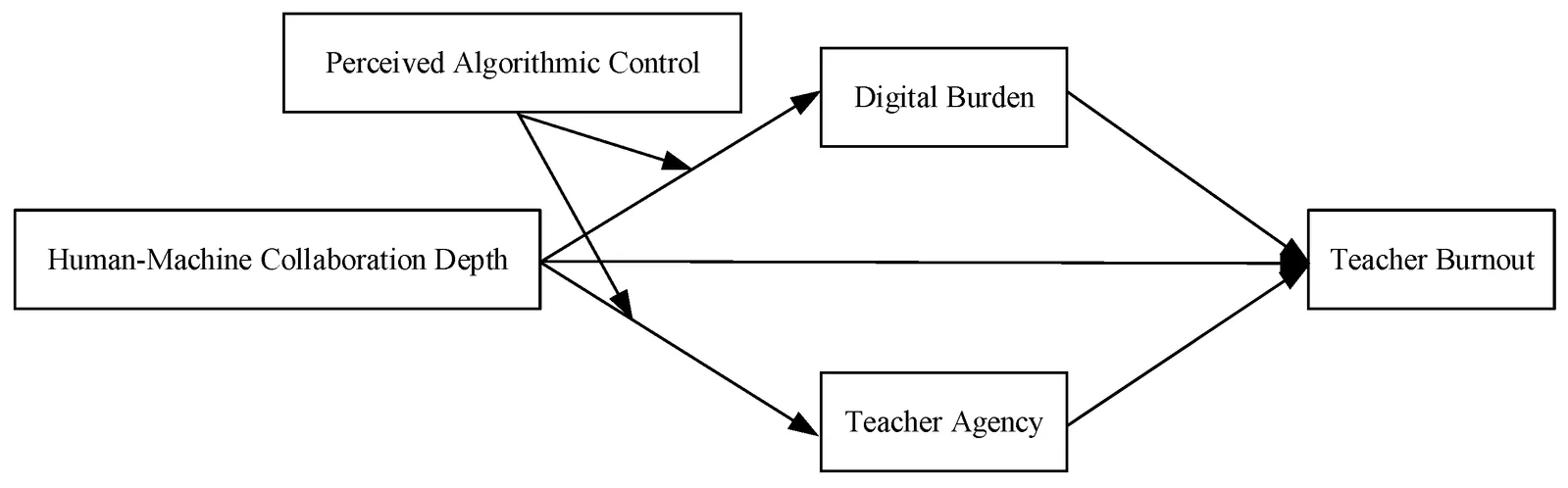
Theoretical Model.

The complete theoretical model encapsulating these hypothesized relationships is presented in Figure 1.

## 3. Materials and Methods

### 3.1. Participants and Procedure

A cross-sectional survey design was used to collect empirical data from full-time university faculty across higher education institutions in mainland China. Strict inclusion criteria were applied to ensure data relevance and quality: participants were required to (1) hold full-time appointments as frontline teaching and research faculty; (2) have a minimum of one year of full-time teaching experience; and (3) have basic familiarity with generative artificial intelligence tools. Part-time instructors and administrative staff were excluded from the sample.

Data were collected via Sojump (Changsha Ranxing Information Technology Co., Ltd., Changsha, China), an online survey platform utilized in behavioral and educational research. A combination of convenience and snowball sampling was employed to maximize sample reach and demographic diversity. The electronic questionnaire was distributed with the assistance of teaching administrators and faculty contacts spanning approximately 17 higher education institutions across 8 provinces. To strictly protect participant anonymity and minimize response bias, specific institutional affiliations were not recorded in the survey. These intermediaries merely forwarded the digital link. The survey interface explicitly guaranteed that participation was entirely voluntary, strictly anonymous, and free from institutional repercussions.

To minimize potential common method variance (CMV), procedural remedies were implemented following the guidelines of Podsakoff et al. (2003). All responses were fully anonymized, and no personally identifiable information was gathered. The order of the survey items was randomized to reduce response set bias, and item wording was simplified to minimize ambiguity.

Before accessing the questionnaire, all respondents reviewed an introductory page detailing the research purpose, data handling protocols, and confidentiality guarantees. Voluntary participation was confirmed via a mandatory informed consent checkbox.

A total of 516 completed questionnaires were received during the data collection period. A rigorous screening process was applied to exclude invalid responses: 27 submissions were removed due to excessively short completion times (<120 s) or straight-lining response patterns, yielding a final analytical sample of 489 valid responses, corresponding to an effective response rate of 94.77%. The final sample size of 489 participants exceeded the minimum threshold recommended for structural equation modeling (Barrett, 2007) and satisfied the 10:1 participant-to-item ratio proposed by Hair et al. (2019), supporting the robustness of the statistical analyses. Notably, 61.96% of respondents reported weekly or daily use of intelligent conversational tools, providing a sound empirical basis for examining technological integration. As summarized in Table 1, the analytical sample exhibits a balanced distribution across academic disciplines—such as STEM (43.76%) and humanities (35.38%)—and professional ranks. This demographic distribution provides broad representation across academic strata, inherently improving sample heterogeneity. Full demographic characteristics are presented in Table 1.

### 3.2. Measures

#### 3.2.1. Scale Adaptation and Content Validity Procedure

The measurement instruments comprised both adapted English scales and an established Chinese scale. Except for teacher burnout, for which a previously validated Chinese version was adopted, all instruments were translated and contextually adapted for the Chinese higher education setting. Standard forward–backward translation procedures (Brislin, 1986) were implemented to ensure semantic equivalence. Rather than intending a formal scale validation, these analyses established measurement adequacy within the present sample. All items utilized a 5-point Likert scale (1 = strongly disagree, 5 = strongly agree), except for teacher burnout, which employed a frequency-based response format. The structural validity was subsequently confirmed via confirmatory factor analysis.

To ensure content validity in the higher education context, the draft questionnaire underwent rigorous expert review prior to pilot testing. Three academic specialists in educational technology and organizational behavior independently evaluated the items for clarity, construct relevance, and contextual appropriateness. Based on their feedback, wording was refined to resolve linguistic ambiguities and improve cultural adaptability. Subsequently, a pilot study was conducted with 76 university instructors to evaluate instrument clarity and psychometric properties. Reliability analyses performed in SPSS 26.0 indicated that all corrected item-total correlations exceeded 0.50, with Cronbach’s *α* coefficients for all latent constructs exceeding 0.80. Minor wording adjustments were integrated based on qualitative feedback, resulting in a final 31-item instrument.

#### 3.2.2. Human–Machine Collaboration Depth

To capture the qualitative nuances of advanced technology utilization, the measure for human–machine collaboration depth was theoretically derived from the system utilization framework of Burton-Jones and Straub (2006). The operationalization of human–machine collaboration depth specifically captures cognitive integration, task sophistication, and creative application. It is conceptually distinct from the mere frequency of technology use (Burton-Jones & Straub, 2006). While frequency measures passive access duration, this construct evaluates the extent of deep interactive dialogue and active pedagogical co-creation. Rather than verbatim item replication, the construct synthesizes the conceptual cores of cognitive absorption and deep structure usage, contextually re-engineered for generative artificial intelligence within higher education. The five-item scale operationalizes human–machine collaboration depth as a cohesive, unidimensional construct that synthesizes user cognition, system functionality, and task integration. Specifically, the items converge to assess high-order feature exploration, deep cognitive engagement, workflow integration, and creative task outputs as interconnected manifestations of a single underlying continuum of deep collaboration. A sample item is “I invest substantial cognitive effort into deep interactions and dialogues with generative AI, rather than simply copying and pasting.” The full set of measurement items for all constructs is provided in Appendix A.

#### 3.2.3. Digital Burden

Technology-induced cognitive and psychological overload among faculty members was measured using a six-item scale derived from the classic technostress framework established by Tarafdar et al. (2007). Instead of adopting the original multidimensional structure, this scale retains the core conceptual essence of three key technostress dimensions: techno-overload, techno-invasion, and techno-complexity. Notably, these six items form a cohesive unidimensional construct that captures the single latent continuum of overall digital burden. Contextual revisions were made to adapt the scale to higher education settings, replacing generic computer-related stressors with specific references to institutional digital platforms and AI educational tools. A representative item reads “The complexity of digital/AI tools increases my workload, requiring significant extra time to learn how to use them.”

#### 3.2.4. Teacher Agency

Teacher agency, defined as the proactive restructuring of professional practices amid technological disruption, was measured using a five-item scale. This scale is theoretically rooted in the technological agency framework developed by Nøhr et al. (2023), which operationalizes agency based on two core dimensions: agentic will and agentic power. To adapt the scale to the context of generative artificial intelligence, item wording was systematically revised by integrating theoretical perspectives from Kahn et al. (2025) and Aagaard et al. (2022). While preserving the core psychological and structural foundations of agency outlined by Nøhr et al. (2023), we expanded the measurement scope to cover collaborative institutional engagement, professional development integration, and proactive pedagogical innovation—key dimensions for adapting to AI-integrated educational environments. A representative item reads “Upon the introduction of technologies like AI, I can proactively redefine my teaching and research methods rather than passively accepting technological arrangements.”

#### 3.2.5. Perceived Algorithmic Control

Perceived algorithmic control, defined as systemic pressure stemming from data-driven quantitative evaluation and automated institutional monitoring, was measured via a six-item scale. This measurement tool is theoretically rooted in the platform tracking and behavioral constraint framework established by Pei et al. (2021) and Zhu et al. (2024). Given that the original scale was developed for gig economy workers, this study conducted rigorous contextual adaptation for higher education settings. We retained the core conceptual dimensions of normative guidance, tracked evaluation, and behavioral constraint, while removing items irrelevant to academic scenarios, such as GPS-based task allocation. All items were contextually adapted to fit institutional digital systems, shifting the measurement focus from physical gig work tasks to the quantitative evaluation of academic outputs. Furthermore, one new item was developed to capture teachers’ perceived absence of human-centric algorithmic management regarding invisible educational labor, including student care work. A representative item reads “I feel the institutional algorithmic management system lacks a human touch, failing to recognize my invisible labor beyond the data (such as care for students).”

#### 3.2.6. Teacher Burnout

Occupational strain, as the core outcome of chronic work-related stress, was evaluated through a nine-item short form derived from the localized Chinese adaptation (Wang et al., 2003) of the Maslach Burnout Inventory (Maslach et al., 2001). To minimize respondent fatigue and ensure psychometric efficiency, we distilled the original 21-item inventory by selecting the highest-loading items for each dimension, thereby retaining the core conceptual essence of emotional exhaustion, depersonalization, and reduced personal accomplishment. The items were contextually re-engineered to reflect the dual demands of teaching and research in higher education; for instance, specific school-level references were broadened to include research-related stressors and colleague interactions, replacing the original K-12 classroom-focused terminology. All nine items were scored on a 7-point frequency scale from 0 (never) to 6 (every day). A sample item is “My work leaves me feeling emotionally drained.”

The Cronbach *α* for all scales in the present study ranged between 0.924 and 0.952, consistently exceeding the critical threshold of 0.70, indicating excellent internal consistency. Detailed results regarding the convergent and discriminant validity of the measurement model are systematically reported in Table 2.

### 3.3. Data Analysis

Quantitative data were processed and analyzed using specialized statistical software. Descriptive statistics and scale reliability were evaluated in IBM SPSS Statistics version 26.0 (IBM Corp., Armonk, NY, USA). Measurement model validation, structural equation modeling, and parallel mediation testing were conducted using AMOS version 24.0 (IBM Corp., Armonk, NY, USA). Furthermore, conditional indirect effects and moderated mediation were tested using Model 7 of the SPSS PROCESS macro version 5.0 (Andrew F. Hayes, Calgary, AB, Canada). Across both parallel and moderated mediation analyses, a bias-corrected bootstrapping procedure with 5000 resamples was applied to determine significance.

## 4. Results

### 4.1. Common Method Bias, Descriptive Statistics, and Construct Validity

Given that all focal constructs were measured via self-report questionnaires, we adopted both procedural and statistical approaches to mitigate potential common method variance (CMV). Procedurally, the survey design guaranteed participant anonymity and randomized item ordering to reduce response bias. Statistically, considering the well-documented limitations of Harman’s single-factor test (Podsakoff et al., 2003), we employed a complementary diagnostic strategy. An unrotated exploratory factor analysis was conducted, and the largest single factor accounted for only 27.213% of the total variance, which is well below the conventional 40% threshold indicative of severe CMV. Furthermore, intercorrelations among all latent constructs remained below the established 0.85 threshold for discriminant validity (see Table 2). These modest correlations confirm the conceptual distinctness of the measured variables. Aligned with the multi-criteria framework of Auh et al. (2016), the available empirical diagnostics indicate that severe common method bias is unlikely to confound the path estimates. Nevertheless, shared method variance cannot be eliminated entirely given the self-reported cross-sectional design (Podsakoff et al., 2003).

To verify the internal consistency, structural calibration, and boundary independence of the measurements, a multi-factor confirmatory factor analysis (CFA) was executed via AMOS 24.0. The overall internal consistency of the constructs was high, with Cronbach’s *α* coefficients ranging tightly between 0.924 and 0.952, exceeding the psychometric reliability requirement of 0.70. For convergent validity evaluation, the standardized item factor loadings ranged from 0.713 to 0.922, establishing high statistical significance (*p* < 0.001). The calculated composite reliability (*CR*) properties spanned from 0.856 to 0.937, while the average variance extracted (*AVE*) calculations ranged from 0.661 to 0.713, satisfying the rigorous benchmarks established by Fornell and Larcker (1981).

Given the multifaceted conceptual design of occupational strain, a secondary higher-order CFA was executed for teacher burnout to validate its operational aggregation. The second-order factor structure displayed excellent fit properties: *χ*^2^/*df* = 1.089, CFI = 0.998, TLI = 0.997, RMSEA = 0.014. The standardized second-order direct paths linking the higher-order burnout construct to its three subordinate dimensions (emotional exhaustion, depersonalization, and reduced personal accomplishment) ranged between 0.691 and 0.863 (*p* < 0.001). This high structural convergence fully justified the aggregation of these sub-dimensions into a singular latent variable for the subsequent correlation and structural path analyses.

Discriminant validity was examined utilizing the Fornell–Larcker criterion, which dictates that the square root of the AVE for any given latent construct must exceed its bivariate correlation with any other parallel construct (Fornell & Larcker, 1981). As mapped along the diagonal in Table 2, the square roots of the *AVE* values (ranging from 0.813 to 0.845) were consistently higher than the corresponding off-diagonal correlation coefficients, thereby confirming robust discriminant validity across the theoretical model. Collectively, these psychometric findings demonstrate robust measurement adequacy and reliability for the adapted instruments within the present sample.

### 4.2. Mediation Effects Testing

The complex indirect linkages mapping the structural model were evaluated through structural equation modeling via AMOS 24.0. Prior to model estimation, statistical assumptions regarding multivariate normality, homoscedasticity, and residual independence were verified. Missing data were managed via listwise deletion during the initial data screening phase, resulting in a complete case dataset. Multicollinearity was evaluated using variance inflation factors, which ranged between 1.357 and 1.626, demonstrating no multicollinearity risks. The comprehensive structural model displayed high empirical alignment and data-model fit indices, namely *χ*^2^/*df* = 1.123, RMSEA = 0.016, CFI = 0.996, TLI = 0.997, and SRMR = 0.041, indicating that the theoretical configurations matched the observed sample correlations.

When both parallel mediators—digital burden and teacher agency—were entered simultaneously, path analysis indicated a non-significant direct link. Specifically, the standardized direct path from human–machine collaboration depth to teacher burnout was not statistically significant (*β* = −0.062, *p* = 0.128). Despite this non-significant direct link, the baseline total effect operated as a significant negative trajectory. Rather than proving causal full mediation, these cross-sectional results suggest an indirect association pattern consistent with a competitive parallel mediation model (MacKinnon & Pirlott, 2015). Specifically, the association between human–machine collaboration depth and teacher burnout is statistically accounted for by two opposing paths.

To unpack and isolate the specific operational transmission dynamics of these concurrent paths, a non-parametric bias-corrected bootstrapping procedure with 5000 resamples was executed. The significance parameters for the specific indirect effects were determined based on whether the computed 95% confidence intervals (*CI*s) excluded zero (Hayes, 2018). As detailed in Table 3, the specific indirect path channeled through the resource-depletion framework of digital burden generated a significant positive effect of 0.095 (Boot *SE* = 0.029, 95% *CI* [0.037, 0.154], *p* < 0.01). This output indicated that an escalation in human–machine collaboration depth is associated with increased cognitive load, which positively relates to teacher burnout. Concurrently, the parallel resource-enrichment path operating via teacher agency produced a highly significant negative indirect effect of −0.229 (Boot *SE* = 0.030, 95% *CI* [−0.292, −0.177], *p* < 0.001), suggesting that human–machine collaboration depth is inversely associated with occupational strain by facilitating professional autonomy.

Furthermore, to empirically distinguish the focal construct from basic technology adoption, the frequency of AI use was specifically included as a covariate along with other demographic factors (e.g., gender, teaching tenure, and academic rank). The main structural relationships and indirect pathways remained highly stable and statistically significant, confirming the unique predictive validity of human–machine collaboration depth beyond simple usage patterns.

In the present sample, the negative indirect pathway via teacher agency (|−0.229|) was numerically larger in magnitude than the positive pathway via digital burden (|0.095|). Consequently, the net total indirect effect remained statistically negative (*β* = −0.134, *p* < 0.01). Although human–machine collaboration depth relates to cognitive strain, its association with teacher agency plays a dominant protective role against teacher burnout. Consequently, the resource gain pathway statistically offsets the resource depletion pathway within the current sample.

### 4.3. Moderation and Moderated Mediation Effects Testing

To evaluate the contextual boundary constraints imposed by the organizational climate, conditional process analysis was conducted using the SPSS PROCESS macro (Model 7) developed by Hayes (2018). Specifically, perceived algorithmic control was posited to moderate the first-stage paths linking human–machine collaboration depth to both digital burden and teacher agency. To accommodate the parallel structure within the regression framework, conditional indirect effects were estimated independently for each mediating pathway. While baseline paths were evaluated using latent variables in AMOS, the regression-based macro was utilized as a complementary tool to decompose conditional indirect effects (Hayes et al., 2017). Observed composite scores were calculated for this analysis, and all focal variables were mean-centered prior to the creation of interaction terms to prevent structural multicollinearity (Hayes, 2018). This procedure controlled for individual demographic variance (gender, teaching tenure, and professional title) and integrated a bias-corrected bootstrapping layout with 5000 resamples to track the interaction mechanics. Statistical indicators for the conditional parameters are detailed in Table 4.

#### 4.3.1. Conditional Interaction Analysis

**Figure 2:**
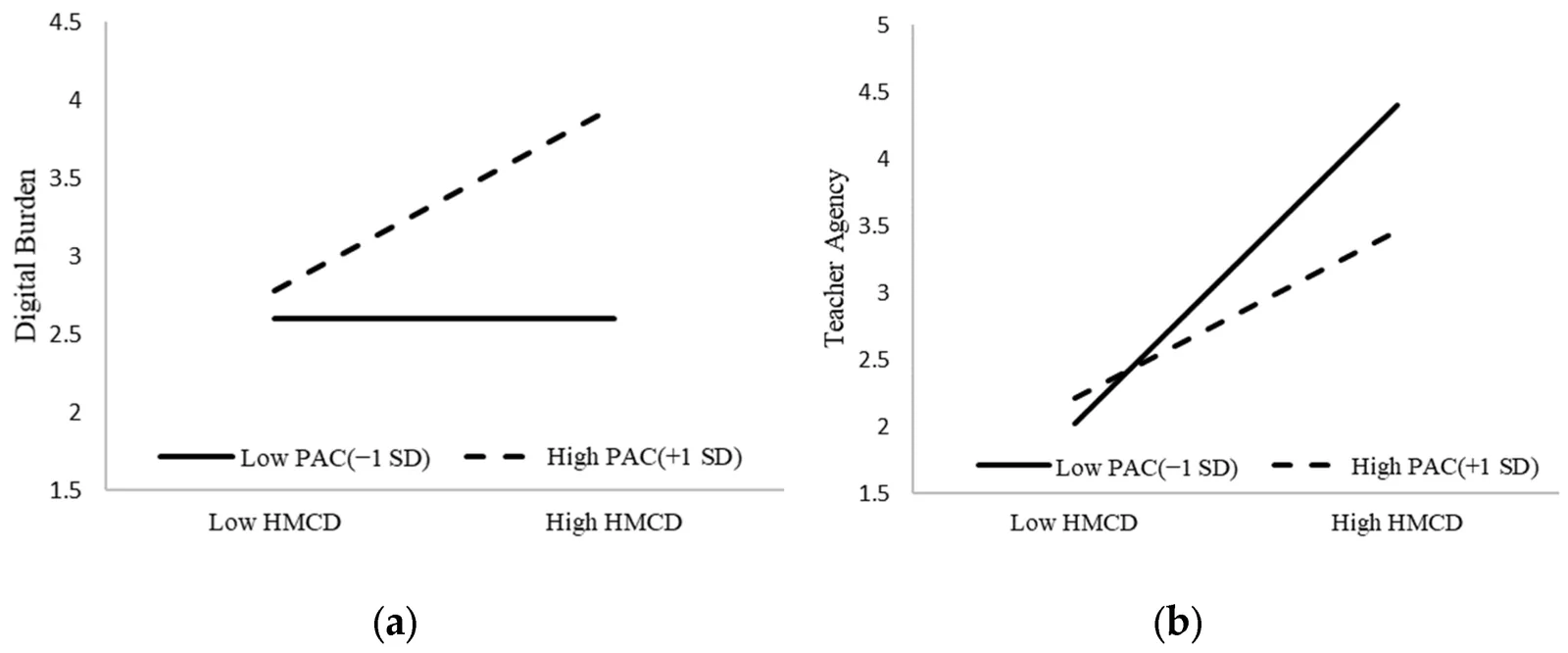
Simple slope plots for the moderating effects of perceived algorithmic control. Notes: (**a**) Interactive effect of HMCD and PAC on DB; (**b**) Interactive effect of HMCD and PAC on TA. Low PAC and High PAC are plotted at −1 *SD* and +1 *SD* from the mean, respectively. HMCD = human–machine collaboration depth; PAC = perceived algorithmic control; DB = digital burden; TA = teacher agency. All focal constructs are mean-centered prior to the conditional process analysis.

Within the resource-depletion trajectory, the product term of human–machine collaboration depth and perceived algorithmic control was a significant positive predictor of digital burden (*B* = 0.147, *p* < 0.001). To map the operational boundary of this interaction, a simple slope analysis was executed at ±1 standard deviation (*SD*) around the moderator mean. Under low perceived algorithmic control (−1 *SD*), the association between human–machine collaboration depth and digital burden was non-significant (*B* = 0.046, *p* = 0.251). However, under high perceived algorithmic control (+1 *SD*), this positive relationship was significantly strengthened (*B* = 0.248, *p* < 0.001). This interaction is illustrated in Figure 2a, with human–machine collaboration depth on the horizontal axis and digital burden on the vertical axis.

Conversely, within the resource-enrichment trajectory, the interaction product term was significantly and negatively related to teacher agency (*B* = −0.141, *p* < 0.001). The corresponding simple slope plots are presented in Figure 2b, maintaining identical horizontal axis mapping for human–machine collaboration depth. When perceived algorithmic control was low (−1 *SD*), deeper human–machine collaboration depth exhibited a strong positive association with teacher agency (*B* = 0.550, *p* < 0.001). Under high perceived algorithmic control (+1 *SD*), this positive association was significantly attenuated, though it maintained a positive slope (*B* = 0.356, *p* < 0.001). This pattern suggests that extensive institutional accountability structures may condition the psychological resource gains derived from technology integration.

#### 4.3.2. Conditional Indirect Path Tracking

To determine whether the overarching transmission efficiency of the dual-path system was systematically altered by the situational moderator, the conditional indirect effect layout and indices of moderated mediation were examined (see Table 4). For the structural path transmitted through digital burden, the computed index of moderated mediation for perceived algorithmic control was established as 0.032, with a bootstrapping 95% confidence interval that completely excluded zero ([0.014, 0.055]). Specifically, under relaxed organizational surveillance (low perceived algorithmic control), the indirect resource-depletion effect was non-significant. However, under high perceived algorithmic control, this indirect trajectory became significant, showing a stronger positive association with teacher burnout (conditional indirect effect = 0.055).

For the parallel path transmitted via teacher agency, the calculated index of moderated mediation for perceived algorithmic control stabilized at 0.022, with the bootstrapping 95% confidence interval excluding zero ([0.007, 0.041]). Under a supportive, low-monitoring climate, the indirect defensive buffer of agency was strong (conditional indirect effect = −0.086). Under automated quantitative tracking (i.e., high perceived algorithmic control), this protective mechanism was weakened (conditional indirect effect = −0.056).

## 5. Discussion

### 5.1. Discussion of Core Findings

Integrating COR theory and SDT, this study tested a moderated mediation model to unpack the psychological mechanisms linking human–machine collaboration depth to teacher burnout across varying levels of perceived algorithmic control.

First, consistent with Hypothesis 1, the structural analysis revealed a significant negative total association between human–machine collaboration depth and teacher burnout. This finding nuances existing discourse on technology-associated strain. While prior research has documented that rapid artificial intelligence integration is primarily linked to educational anxiety and psychological exhaustion (Zhang & Cao, 2025), the present study identifies a competing dynamic. Specifically, resource gains tied to deep technological synergy may counterbalance initial adaptation stress. A comparison of indirect pathways showed that the empowerment pathway via teacher agency was numerically larger in magnitude than the depletion pathway via digital burden within this sample. This asymmetric pattern aligns with recent evidence indicating that preserved professional autonomy during human–AI interaction represents a key protective mechanism against workplace fatigue (Kahn et al., 2025).

Second, the findings are consistent with a full statistical mediation of the association between human–machine collaboration depth and teacher burnout by digital burden and teacher agency. When both mediators were entered into the model, the direct link between human–machine collaboration depth and teacher burnout became non-significant. This pattern of statistical mediation runs counter to a technological determinist perspective: AI integration does not inherently determine teacher well-being. Rather, its psychological correlates vary depending on how human–AI interaction is internalized. When engagement with technology remains at the level of superficial task execution, it is positively associated with resource loss via digital burden (Tarafdar et al., 2007). This pattern is consistent with Hypothesis 2. Conversely, when such integration translates into greater pedagogical competence and decision-making authority, it is negatively associated with burnout through the potential replenishment of core psychological resources. This pattern is consistent with Hypothesis 3.

Third, the moderation analysis confirmed perceived algorithmic control as a critical organizational boundary condition. Higher perceived algorithmic control altered the relational profile of technological engagement. Specifically, more intensive digital surveillance was associated with a stronger positive indirect association between collaboration depth and burnout via digital burden (consistent with Hypothesis 4), aligning with prior work indicating that automated evaluation systems are tied to elevated work intensity (Röhl, 2025). At the same time, greater monitoring was linked to a weaker protective empowerment pathway via teacher agency (consistent with Hypothesis 5), dampening the translation of technological synergy into professional autonomy. Taken together, the flexibility of the external managerial environment shapes the conditions under which intelligent educational tools relate to psychological outcomes.

### 5.2. Theoretical Implications

This study makes three theoretical contributions to the literature on occupational health psychology and intelligent education.

First, it nuances the technology–strain nexus by moving the theoretical dialogue beyond a binary opposition toward a competitive integration perspective. Prior scholarship has often linked disruptive digital environments to technological anxiety and subsequent burnout among knowledge workers (Högemann et al., 2025). We extend this work by suggesting that the depleting effects associated with technology are most evident during superficial adaptation phases or under conditions of high surveillance. By identifying the dominance of the empowerment pathway, this study expands the boundary conditions of existing technology acceptance frameworks, suggesting that deep cognitive synergy may act as a protective factor against technology-associated strain.

Furthermore, rather than demonstrating dynamic processes, our cross-sectional findings offer empirical patterns consistent with the integration of COR theory and SDT. While the prior technostress literature emphasizes energetic depletion from information overload (Tarafdar et al., 2007), our model delineates distinct theoretical boundaries for the parallel pathways. Specifically, digital burden captures a COR-grounded resource drain, whereas teacher agency represents an SDT-guided motivational asset. Although cross-sectional data cannot directly confirm dynamic resource spirals, the results provide a plausible static configuration of concurrent empowerment and depletion.

Finally, this research extends the application of algorithmic management theory from the gig economy (Zhu et al., 2024) to the professionalized higher education context. While prior work has described how platform algorithms shape the physical labor of gig workers, this study suggests that automated quantitative evaluation processes are linked to the erosion of intellectual autonomy among university faculty. The data are consistent with the view that rigid algorithmic governance is associated with a crowding-out effect on intrinsic pedagogical motivation, potentially limiting the empowerment potential of human–machine synergy and sustaining a cycle of quantitative compliance among educators.

### 5.3. Practical Implications

Building on the observed associational patterns, this study offers three practical implications for higher education administrators to advance sustainable educational digital transformation.

First, because teacher agency exhibits a negative association with teacher burnout, faculty development initiatives should prioritize professional autonomy. Rather than solely offering operational tutorials, institutions might explore applications such as cultivating AI literacy. Training programs could empower faculty to autonomously redesign curricula and critically evaluate AI outputs (Dehghan, 2026). By structurally supporting pedagogical control, universities may enhance psychological resilience against occupational exhaustion.

Second, given that digital burden is positively associated with teacher burnout, institutions should actively minimize unnecessary digital overload. Potential strategies derived from this finding include establishing targeted technical support structures. For instance, dedicated IT helpdesks or curated prompt-engineering repositories might reduce initial cognitive friction. Furthermore, establishing clear policies regarding acceptable digital availability may prevent work technology from encroaching into personal recovery time (Högemann et al., 2025; Tarafdar et al., 2007).

Third, because perceived algorithmic control weakens the resource gain pathway, evaluation systems should avoid excessive surveillance. To mitigate this boundary effect, administrators might reduce their reliance on rigid quantitative metrics. Specific applications could involve interpreting platform-generated metrics, such as login duration, with greater caution. Incorporating formative evaluation criteria, including peer review, helps position algorithms as diagnostic tools rather than punitive surveillance mechanisms (Kellogg et al., 2020; Zayid et al., 2024).

### 5.4. Limitations and Future Research

Several limitations should be acknowledged to guide future research. First, utilizing a cross-sectional survey design precludes definitive causal inferences (Spector, 2019), as it cannot fully capture the shifting temporal dynamics between human–machine collaboration depth and teacher burnout. Second, reliance on self-report measures and convenience sampling introduces inherent boundaries. Despite rigorous procedural and statistical controls, these cross-sectional and self-reported designs preclude the absolute elimination of shared method variance (Podsakoff et al., 2003). Additionally, potential self-selection bias may exist among educators highly interested in artificial intelligence tools. Third, our empirical evaluation lacked objective data regarding actual artificial intelligence usage metrics and organizational-level parameters on real algorithmic infrastructures. To address these constraints, future research should employ time-lagged designs or multi-source evaluations, such as objective administrative data, to validate the associations within the model (Zhu et al., 2024).

Fourth, potential reverse causality may operate within the model. Individual educators with lower baseline teacher burnout or elevated teacher agency might actively pursue human–machine collaboration depth. From a resource perspective, this dynamic may signify a reciprocal gain spiral, a path requiring longitudinal tracking (Hobfoll, 2001). Fifth, the empirical sample was restricted to Chinese higher education institutions. The distinct institutional culture may condition the baseline manifestation of perceived algorithmic control. Finally, perceived algorithmic control might partially intercept broader digital performance pressure or managerial surveillance. In contemporary universities, algorithmic metrics are intrinsically embedded within quantitative evaluation infrastructures. Subsequent studies should employ cross-cultural designs to disentangle automated algorithmic management from general organizational stressors. Additionally, incorporating unexamined protective moderators like technological self-efficacy (Zivi et al., 2025) could further refine these predictive boundaries.

## 6. Conclusions

Greater human–machine collaboration depth was associated with lower teacher burnout through competing indirect pathways involving digital burden and teacher agency. Within this cross-sectional framework, the resource gain pathway statistically offset the resource depletion pathway. Furthermore, perceived algorithmic control conditioned these associations, strengthening the indirect strain pathway and constraining professional autonomy. As this study relies on self-reported survey data, these structural relationships require confirmation through longitudinal, experimental, or multi-source designs. Ultimately, the psychological outcomes of technology integration are conditionally associated with institutional evaluation structures alongside the digital tools themselves. Addressing occupational strain therefore calls for balanced governance systems that reconcile data-driven metrics with educator autonomy.

## Figures and Tables

**Table 1:** Demographic characteristics of the participants (*n* = 489).

Variable	Category	Frequency	Percentage (%)
Gender	Male	228	46.63
	Female	261	53.37
Disciplinary Background	Humanities and Social Sciences	173	35.38
	Science, Engineering, Agriculture, and Medicine	214	43.76
	Arts, Sports, and Others	102	20.86
Frequency of AI Use	Occasionally	186	38.04
	Weekly	182	37.22
	Daily	121	24.74
Years of Teaching Experience	<3 years	36	7.36
	3–5 years	107	21.88
	6–10 years	151	30.88
	11–20 years	106	21.68
	>20 years	89	18.2
Academic Rank	Teaching Assistant	65	13.29
	Lecturer	249	50.92
	Associate Professor	126	25.77
	Professor	49	10.02

**Table 2:** Descriptive statistics, reliability, and convergent/discriminant validity matrix (*n* = 489).

	Mean	*SD*	Cronbach’s *α*	*CR*	*AVE*	HMCD	DB	TA	PAC	TB
HMCD	2.971	0.661	0.936	0.906	0.661	**0.813**				
DB	2.982	0.653	0.946	0.929	0.686	0.153 **	**0.828**			
TA	3.007	0.645	0.936	0.925	0.713	0.468 **	−0.110 *	**0.844**		
PAC	2.995	0.682	0.952	0.937	0.714	0.027	0.585 **	−0.265 **	**0.845**	
TB	2.980	0.370	0.924	0.856	0.667	−0.180 **	0.541 **	−0.470 **	0.444 **	**0.817**

Note: Bold values along the diagonal represent the square root of the *AVE*. Off-diagonal metrics represent standardized Pearson correlation coefficients. HMCD = human–machine collaboration depth; DB = digital burden; TA = teacher agency; PAC = perceived algorithmic control; TB = teacher burnout. * *p* < 0.05, ** *p* < 0.01.

**Table 3:** Structural path coefficients and bootstrapping effect decomposition results (*n* = 489).

Structural Paths/Effect Pathways	Standardized Estimate	*SE*	*p*-Value	95% *CI* (*LL*, *UL*)
Direct Structural Paths (*β*)				
HMCD → TB	−0.062	0.040	0.128	–
HMCD → DB	0.152	0.045	**	–
DB → TB	0.626	0.029	***	–
HMCD → TA	0.499	0.040	***	–
TA → TB	−0.459	0.039	***	–
Effect Decomposition				
Total Effect	−0.196	0.048	***	[−0.286, −0.096]
Direct Effect	−0.062	0.040	0.128	[−0.140, 0.017]
Total Indirect Effect	−0.134	0.045	**	[−0.222, −0.045]
HMCD → DB → TB (Loss Path)	0.095	0.029	**	[0.037, 0.154]
HMCD → TA → TB (Gain Path)	−0.229	0.030	***	[−0.292, −0.177]

Note: HMCD = human–machine collaboration depth; DB = digital burden; TA = teacher agency; TB = teacher burnout. *SE* = Standard Error; *CI* = Confidence Interval from 5000 bootstrap resamples. Gender, disciplinary background, frequency of AI use, years of teaching experience and academic rank were controlled as covariates in the structural model. → = structural path or directional relation; – = not applicable. ** *p* < 0.01, *** *p* < 0.001.

**Table 4:** Conditional process estimation and moderated mediation outputs (*n* = 489).

Constructs and Pathways	Value	*SE*	*t*-Value	95% *CI* (*LL*, *UL*)
First-Stage Interaction Effects	*B*			
HMCD × PAC → DB	0.147	0.039	3.771 ***	[0.070, 0.224]
Conditional Effects at PAC = −1 *SD*	0.046	0.04	1.150	[−0.032, 0.124]
Conditional Effects at PAC = +1 *SD*	0.248	0.045	5.512 ***	[0.159, 0.337]
HMCD × PAC → TA	−0.141	0.040	−3.530 ***	[−0.220, −0.062]
Conditional Effects at PAC = −1 *SD*	0.550	0.046	11.951 ***	[0.460, 0.640]
Conditional Effects at PAC = +1 *SD*	0.356	0.050	7.123 ***	[0.258, 0.454]
Conditional Indirect Effects	Indirect Effect/Index	Boot *SE*		
HMCD → DB → TB (Loss Path)				
Index of Moderated Mediation	0.032	0.010	–	[0.014, 0.055]
Conditional Indirect Effect at PAC = −1 *SD*	0.010	0.011	–	[−0.006, 0.028]
Conditional Indirect Effect at PAC = +1 *SD*	0.055	0.015	–	[0.029, 0.085]
HMCD → TA → TB (Gain Path)				
Index of Moderated Mediation	0.022	0.008	–	[0.007, 0.041]
Conditional Indirect Effect at PAC = −1 *SD*	−0.086	0.014	–	[−0.116, −0.057]
Conditional Indirect Effect at PAC = +1 *SD*	−0.056	0.012	–	[−0.081, −0.033]

Note: *B* = unstandardized regression coefficient. HMCD = human–machine collaboration depth; DB = digital burden; TA = teacher agency; PAC = perceived algorithmic control; TB = teacher burnout. Control variables include gender, disciplinary background, frequency of AI use, years of teaching experience and academic rank. *LL* = Lower Limit, *UL* = Upper Limit, *CI* = Confidence Interval from 5000 bootstrap resamples. → = structural path or directional relation; – = not applicable. *** *p* < 0.001.

## Data Availability

The data presented in this study are available from the corresponding author upon request.

## Abbreviations

The following abbreviations are used in this manuscript:

GenAI	Generative artificial intelligence
HMCD	Human–machine collaboration depth
DB	Digital burden
TA	Teacher agency
TB	Teacher burnout
PAC	Perceived algorithmic control
COR	Conservation of Resources
SDT	Self-determination theory

## Appendix A

**Table A1:** Measurement items.

**Human–Machine Collaboration Depth (Adapted from Burton-Jones & Straub, 2006)**
1. I have deeply integrated generative AI into my daily instructional design or research workflow, making it an indispensable part of my work.
2. I rely heavily on the support provided by generative AI when handling complex teaching or research tasks.
3. I frequently explore and experiment with advanced features of generative AI (e.g., complex prompt engineering, multi-turn iterative dialogues) rather than just using basic functions.
4. I can utilize generative AI in a creative manner, enabling outputs that exceed conventional expectations.
5. I invest substantial cognitive effort into deep interactions and dialogues with generative AI, rather than simply copying and pasting.
**Digital Burden (Adapted from Tarafdar et al., 2007)**
1. To adapt to constantly updated digital/AI tools, I am forced to accelerate my work pace, leading to an overloaded workload.
2. I am forced to change my customary working methods to adapt to various digital/AI systems introduced in my institution.
3. The complexity of digital/AI tools increases my workload, requiring significant extra time to learn how to use them.
4. Digital tools (e.g., ubiquitous mobile work) blur the boundaries between my work and family life, making me feel constantly on call.
5. Due to the convenience of digital tools, I feel tethered to my work, finding it difficult to fully detach even during holidays.
6. The massive amount of information from various digital channels and AI-generated content leaves me feeling overwhelmed and anxious.
**Teacher Agency (Adapted from Nøhr et al., 2023, with conceptual extensions grounded in Kahn et al., 2025 and Aagaard et al., 2022)**
1. Upon the introduction of technologies like AI, I can proactively redefine my teaching and research methods rather than passively accepting technological arrangements.
2. I possess sufficient voice and decision-making power regarding how AI technology is applied to my professional processes.
3. I can clearly identify the significance of technology for my professional development and integrate it with my educational philosophy.
4. I actively participate in institutional discussions regarding technology application and contribute my professional insights.
5. I am not merely a user of technology, but an active improver and developer within the context of technology application.
**Perceived Algorithmic Control (Adapted from Pei et al., 2021; Zhu et al., 2024)**
1. The institutional digital systems/AI platforms provide standardized processes and norms for my work that I must strictly follow.
2. I feel that the institutional digital systems are recording and tracking my work traces in real-time (e.g., online teaching duration, system logins).
3. The institution overly relies on algorithm-generated quantitative metrics (e.g., KPIs, citation rates) to evaluate my work performance.
4. The system automatically generates feedback or rankings based on my data performance, creating invisible pressure for me.
5. My evaluations or performance bonuses are directly affected if I fail to meet certain metrics or requirements set by the digital systems.
6. I feel the institutional algorithmic management system lacks a human touch, failing to recognize my invisible labor beyond the data (such as care for students).
**Teacher Burnout (Adapted from Wang et al., 2003)**
1. I feel exhausted at the end of the workday.
2. I feel burned out from my work.
3. My work leaves me feeling emotionally drained.
4. I have become increasingly callous and indifferent toward others (e.g., students or colleagues).
5. I find myself treating students or work tasks mechanically, like inanimate objects.
6. I sometimes do not really care what happens to my students, focusing only on task completion.
7. I can effectively solve problems that arise in my teaching or research. (Reverse scored)
8. I feel I am positively influencing other people’s lives through my work. (Reverse scored)
9. I feel I am making valuable contributions to the growth of my school or students. (Reverse scored)
